# Inflammatory and Bone Remodeling Responses to the Cytolethal Distending Toxins

**DOI:** 10.3390/cells3020236

**Published:** 2014-04-04

**Authors:** Georgios N. Belibasakis, Nagihan Bostanci

**Affiliations:** 1 Oral Microbiology and Immunology, Institute of Oral Biology, Center of Dental Medicine, University of Zürich, Plattenstrasse 11, Zürich 8032, Switzerland; 2 Oral Translational Research, Institute of Oral Biology, Center of Dental Medicine, University of Zürich, Plattenstrasse 11, Zürich 8032, Switzerland; E-Mail: Nagihan.Bostanci@zzm.uzh.ch

**Keywords:** cytolethal distending toxin, cytokine, inflammation, bone remodeling, immune response, *Aggregatibacter actinomycetemcomitans*, *Haemophilus ducreyi*, *Campylobacter jejuni*, *Helicobacter hepaticus*

## Abstract

The cytolethal distending toxins (CDTs) are a family of exotoxins produced by a wide range of Gram-negative bacteria. They are known for causing genotoxic stress to the cell, resulting in growth arrest and eventually apoptotic cell death. Nevertheless, there is evidence that CDTs can also perturb the innate immune responses, by regulating inflammatory cytokine production and molecular mediators of bone remodeling in various cell types. These cellular and molecular events may in turn have an effect in enhancing local inflammation in diseases where CDT-producing bacteria are involved, such as *Aggregatibacter actinomycetemcomitans*, *Haemophilus ducreyi*, *Campylobacter jejuni* and *Helicobacter hepaticus*. One special example is the induction of pathological bone destruction in periodontitis. The opportunistic oral pathogen *Aggregatibatcer actinoycemetemcomitans*, which is involved in the aggressive form of the disease, can regulate the molecular mechanisms of bone remodeling in a manner that favors bone resorption, with the potential involvement of its CDT. The present review provides an overview of all known to-date inflammatory or bone remodeling responses of CDTs produced by various bacterial species, and discusses their potential contribution to the pathogenesis of the associated diseases.

## 1. Introduction

The family of cytolethal distending toxins (CDTs) consists of a number of bacterial protein exotoxins expressed by a broad range of Gram-negative bacteria, with a potential involvement in the pathogenesis of a diverse range of human infections. They can be described as genotoxins, as their main action is to elicit DNA damage responses on the intoxicated host cells. Consequently, their action leads to cell cycle arrest and eventually cell apoptosis [1,2]. The CDT holotoxin consists of three subunits, namely CdtA, CdtB and CdtC. The CdtA and CdtC subunits mediate the internalization of the CdtB subunit into the cell, which is a molecule functionally homologous to deoxyribonuclease I. It is therefore CdtB which is responsible for the induction of these deleterious effects on the host cells. Apart from these well-characterized effects of CDTs, the literature indicates that CDTs may also be involved on other aspects of the pathogenesis of infection, such as the establishment of a chronic inflammatory response [3,4]. It is therefore the aim of this review to identify and present the current state of the literature with regards to the role of CDTs on host inflammatory responses, as well as the associated effects on bone remodeling.

## 2. Host Inflammatory Responses to Cytolethal Distending Toxins

Inflammation is a process that is triggered in response to a noxious stimulus, such as a bacterial pathogen or its virulence factors. Many bacterial virulence factors can act as pathogen-associated molecular patterns (PAMPs), which are highly conserved bacterial structures. Pattern recognition receptors (PRRs) are transmembrane or intracellular receptors of eukaryotic cells, specialized in the recognition of PAMPs. Therefore, PRRs are responsible sensing and responding to the invading bacteria. Examples of PRRs are the network of Toll-like (TLRs), nucleotide-binding oligomerization domain (NODs) and nod-like receptors (NLRPs) [5]. Activation of PRRs results in the maturation and extracellular secretion of cytokines, such as interleukins (ILs), that can thereafter exert their biological roles [6]. Cytokines are a group of diverse molecules that mediate the cell-to-cell signaling during the initiation and establishment of inflammation. As the CDTs are well established virulence factors with potential pro-inflammatory actions upon host cells, they can certainly classify as PAMPs. The effects of CDT on inflammation may not be necessarily restricted to a particular member of this toxin family. Accordingly, various host cell types are responsive to CDTs, in terms of host inflammatory responses. This section summarizes the inflammatory responses of host cells to CDT, according to each specific member of this toxin family. The literature search yielded pertinent publications in the cases of *Aggregatibacter actinomycetemcomitans*, *Haemophilus ducreyi*, *Campylobacter jejuni* and *Helicobacter hepaticus*. A summary of the associated effects and corresponding literature is provided in Table 1.

### 2.1. Effect of Aggregatibacter actinomycetemcomitans CDT

*Aggregatibacter actinomycetemcomitans* is a Gram-negative facultative anaerobe, which is highly implicated in the pathogenesis of localized aggressive periodontitis, a disease leading to tooth loss in adolescents or young adults. This species expresses two protein exotoxins, namely a leukotoxin and a CDT, which is a unique property of *A. actinomycetemcomitans*, among all known oral species [7,8]. While the CDT of *A. actinomycetemcomitans* has been shown to exert its “classical” growth inhibitory effects in human T-lymphocytes [9], B-lymphocytes [10], mononuclear cells [11], epithelial cells [11,12], gingival fibroblasts and periodontal ligament cells [13,14], there is also evidence that this toxin is involved in the stimulation of cytokine production by the host.

The first indication on such an effect came from the study of Akifusa [11]. This study used recombinant *A. actinomycetemcomitans* CDT holotoxin to challenge isolated human peripheral blood mononuclear cells (PBMCs), and identified that it was able to induce the synthesis of IL-1β, IL-6, and IL-8, but not tumor necrosis factor (TNF)-α, IL-12, or granulocyte-macrophage colony-stimulating factor (GM- CSF). When the different subunits were tested individually in this experimental system, CdtC appeared to be a more potent inducer of these cytokines, compared to CdtB, indicating that cytokine-inducing action is independent of the deoxyribonuclease I activity of the toxin (which is conferred by CdtB). A synergistic cytokine-inducing capacity was also demonstrated, most markedly in the case of interferon (IFN)-γ. None of the individual subunits demonstrated IFN-γ-stimulating capacity, whereas higher concentrations of the combination of CdtB and CdtC, and particularly all three subunits together, demonstrated a strong stimulatory effect. The three primary cytokines shown to be induced in PBMCs by *A. actinomycetemcomitans* CDT are of pro-inflammatory nature. Hence their induction is commensurate with the inflammatory traits of periodontal diseases. IL-8 in particular is a major chemokine, whose presence is responsible for recruiting neutrophils in the affected area, hence intensifying the histopathological events of inflammation [15]. IL-1β and IL-6 are also consider osteolytic cytokines, with a primary role in osteoclast formation and subsequent bone resorption [16], which is the hallmark of periodontitis. Hence, their induction by may denote an effect on the initiation of inflammation in the periodontal tissues that could lead to the pathological process of periodontal tissue breakdown, and hence periodontitis.

The effect of *A. actinomycetemcomitans* CDT has also been investigated on the production of nitric oxide (NO) by murine peritoneal macrophages [17]. NO is a free radical and a crucial inflammatory mediator. It was found that CDT caused a rapid inhibition of NO production by these cells, which was directed towards IFN-γ-dependent inflammatory pathways, and was independent of anti-inflammatory cytokines IL-4 or IL-10. This was interpreted as an immunosuppressive effect of CDT, beyond its growth inhibitory and apoptotic capacities, which could be involved in the pathogenesis of *A. actinomycetemcomitans*–associated periodontitis. Hence, inhibition of NO production in macrophages by *A. actinomycetemcomitans* CDT could putatively hinder a cascade of pro-inflammatory events that lead to elimination of the invading bacterial pathogens, favoring the establishment of chronic periodontal infection.

Apart from cells of the immune system, structural cells including gingival fibroblasts (GF) and periodontal ligament (PDL) cells have been studied for their inflammatory responses to CDT. By the use of CDT-deletion mutants, it was found that, although *A. actinomycetemcomitans* induced IL-6, IL-1β and TNF-α gene expression in GF, the CDT was actually not in involved in these transcriptional events. Nevertheless, rather than transcription, IL-6 cytokine secretion was stimulated by *A. actinomycetemcomitans* challenge, and CDT conferred a partial additive effect to this capacity. On the contrary, there was no difference in IL-1β or TNF-α secretion. The gene expression of the different receptor subtypes for IL-6, IL-1β and TNF-α cytokines was also not affected by *A. actinomycetemcomitans*, either this expressed CDT or not [18]. Therefore, *A.actinomycetemcomitans* induces IL-6 production in GF, albeit with only partial involvement of the CDT. Accordingly, in another study it was shown that soluble surface extracts from *A. actinomycetemcomitans* cells stimulate the production of several pro-inflammatory cytokines (including IL-1β, TNF-α, IL-6, IL-8, MIP-1β) by whole human blood, but the CDT or the leukotoxin were not crucial in this respect, as demonstrated also by the use of a double mutant for these two toxins [19].

Of interest is also a study on inflammasome expression in human mononuclear cells (MNLs), in response to *A. actinomycetemcomitans*[20]. Inflammasomes are intracellular complexes that are activated by the PAMP-PRR interaction, responsible for the maturation and release of IL-1 cytokines by cells of the immune system [21]. It was shown that *A. actinomycetemcomitans* does not affect the expressions of NLRP1, NLRP2 and Absent In Melanoma (AIM)2 inflammasome sensors, or their effector Caspase-1. Nevertheless, it does cause an up-regulation of the NLRP3 and a down-regulation of the NLRP6 sensor. This effect was not dependent on the leukotoxin or the CDT, as demonstrated by the use of the CDT-deletion strains. Hence, the capacity of *A. actinomycetemcomitans* to regulate the expression of inflammasomes does not appear to be attributed to its CDT [20].

The nature on the host cell receptor via which *A. actinomycetemcomitans* CDT exerts its actions is not clear. Yet, there are indications that the glycosphingolipid GM3 can act as one [22,23]. The receptor binding capacity of CDT is attributed to the aromatic aminoacids of the CdtA subunit [24,25], but not restricted to these [26].

Collectively, *A. actinomycetemcomitans* components other than the CDT appeared to be stronger regulators of pro-inflammatory cytokines in the various experimental systems. However, the cytokine-inducing capacity of *A. actinomycetemcomitans* CDT appears to be evident in PBMCs and GF, particularly in the case of IL-6, a cytokine with a pronounced role in the stimulation of bone resorption [27]. Apart from the regulation of IL-6, the potential effects of CDT on bone resorption are further discussed in the next section.

### 2.2. Effect of H. ducreyi CDT

*Haemophilus ducreyi* is a Gram-negative species that is the etiological factor of chancroid, a disease characterized by genital ulcers and regional lymphadenitis. It expresses a CDT which is highly homologous to that of *A. actinomycetemcomitans*, exhibiting 96% amino-acid sequence identity. For this reason, it has also been used in its purified form as a model CDT in experimental systems relevant to *A. actinomycetemcominans* and periodontal pathogenesis. In one such example, purified *H. ducreyi* CDT was able to induce the secretion, but not transcription of IL-6 by GF [18]. Accordingly, it has also been shown that normal cells, as well as cancer cell lines, intoxicated with *H. ducreyi* CDT were induced to express several cytokines, particularly IL-6, IL-8 and IL-24 [28]. Nevertheless, in another *in vitro* study, purified *H. ducreyi* CDT partially inhibited the production of TNF-α, IL-6, IL-8, and IL-12 by monocyte-derived dendritic cells, thus hampering the early stage immune responses to *H. ducreyi* [29]. Hence, although there is still limited volume of literature on this theme, *H. ducreyi*CDT may stimulate or inhibit cytokine production, depending on the experimental cell system employed.

### 2.3. Effect of Campylobacter jejuni CDT

*Campylobacter jejuni* is a Gram-negative species associated with enterocolitis, a local acute inflammatory response that involves intestinal tissue damage and diarrhea. The intestinal epithelium is a physical barrier that responds to *C. jejuni* infection by eliciting an inflammatory response. *C. jejuni* is yet another species that expresses a potent CDT, which can modulate the host inflammatory responses of the gut epithelium. An early study using intestinal epithelial cells has shown that C. jejuni can induce IL-8 release, attributed to a large extent to its CDT. Indeed, anti-CDT antibodies neutralized its capacity to stimulate IL-8 release [30]. In other studies involving human intestinal epithelial cells, it was confirmed that infection with *C. jejuni* or just its outer membrane vesicles, was able to stimulate the production of pro-inflammatory IL-8, as well as the anti-inflammatory cytokine IL-10. However, CDT-deficient strains elicited similar effects, suggesting a CDT-independent mode of action [31,32]. When the effect of *C. jejuni* challenge was considered on human colonic epithelial cells, both IL-8 and TNF-α cytokines were up-regulated. Its ability to stimulate IL-8 secretion was reduced when CDT-deletion strains were used, indicating a crucial role of CDT here, which was also dependent on the activation of NF-κB and toll-like receptor (TLR) signaling [33]. It should be reminded that transcription factor NF-κB activates a number of pro-inflammatory cytokine genes, including IL-1β, IL-6, IL-8 and TNF-α [34]. Therefore, it possesses a key role in the initiation of the inflammatory events in response to bacterial challenge. In an interaction model using human monocytic cells, *C. jejuni* was able to induce a number of pro-inflammatory cytokines, including IL-1α, IL-1β, IL-6, IL-8, and TNF-α, but CDT was not involved [35,36]. Collectively, there is some evidence that the CDT of *C. jejuni* stimulates IL-8 production by gut epithelial cells, which may promote neutrophil migration and inflammatory events in the intestinal tissue.

### 2.4. Effect of Helicobacter hepaticus CDT

*Helicobacter hepaticus* is a Gram-negative CDT-producing species, associated with chronic gastrointestinal inflammation and neoplasia. It is also involved in the development of typhlocolitis and chronic hepatitis. The studies available in the literature regarding the inflammatory effects of *H. hepaticus* CDT are primarily using animal infection models, rather than *in vitro* models. Challenge of IL-10(−/−) mice with isogenic *H. hepaticus* mutants showed that CDT is not required for colonization of the murine gut. Despite this, a CDT-deficient mutant significantly diminished the capacity of *H. hepaticus* to induce lesions in this murine model of inflammatory bowel disease [37]. In line with these findings, both a wild-type *H. hepaticus* and its derivative CDT-deficient mutant successfully colonized the intestinal tissue of IL-10(−/−) mice. Still, only the wild-type infected mice developed eventually typhlocolitis. However, at longer periods the CDT-deficient mutant could not be detected any further in the mice, but the wild-type strain could persist for longer periods [38]. In line with this finding, in another study using an isogenic *H. hepaticus* CDT-deficient mutant to infect Swiss Webster mice it was shown that the presence of the toxin was important for longer term colonization of this species, and associated with down-regulation of colonic IL-10 production [39]. In yet another study, infection with an isogenic *H. hepaticus* CDT mutant induced chronic hepatitis in mice. The pathological traits were comparable to those elicited by the wild-type *H. hepaticus*, with the exception that the latter caused additionally hepatic dysplasic nodules. Moreover, the wild-type infected mice demonstrated significantly enhanced hepatic tissue expression of TNF-α, IFN-γ and IL-6 [40]. Collectively, it appears that *H. hepaticus* CDT is not required for intestinal colonization, but is important for eliciting the chronic inflammatory response associated with colitis, and some of the histopathological traits of colitis and hepatitis.

## 3. Bone Remodeling Responses to Cytolethal Distending Toxins

Special mention should be made to the potential of CDT to perturb bone metabolism, particularly by interfering in the signaling for osteoclast differentiation. Among all CDT-producing species, it is the oral pathogen *A. actinomycetemcomitans* for which this virulence property could be of relevance. That is because periodontitis, in which *A. actinomycetemcomitans* is involved as a putative pathogen, pertains bone destruction of the tooth-supporting tissue. Hence, periodontitis is the only bone-related pathology in which a CDT family member may be involved.

Receptor activator of NF-κB ligand (RANKL) is a membrane-bound or soluble ligand expressed by osteoblasts, fibroblasts and activated T-cells and B-cells. By binding onto its cognate RANK receptor on the surface of pre-osteoclasts, it triggers their differentiation into mature osteoclasts, which are the cells responsible for bone resorption. On the contrary, osteoprotegerin (OPG) is a soluble decoy receptor, responsible for binding to RANKL and therefore for blocking its action. This system of molecules is crucial for bone resorption, and the relative RANKL/OPG ratio is shown to be elevated in bone-destructive periodontal disease [41,42,43,44].

The current literature related to the RANKL-OPG system and *A. actinomycetemcomitans* CDT is pertinent to *in vitro* experimental systems. *A. actinomycetemcomitans* was able to induce RANKL gene expression, as well as cell-membrane RANKL protein up-regulation, in GF and PDL cells, which are both structural cells of the periodontium. However, OPG gene expression or secretion remained unaffected. An *A. actinomycetemcomitans* CDT-deletion mutant was not able to induce RANKL expression, in contrast to its parental wild-type. In agreement with this finding, pretreatment of *A. actinomycetemcomitans* wild-type with antisera raised against CDT, abolished RANKL expression [45]. Inhibition of IL-6, IL-1, TNF-α or prostaglandin E_2_, which are all well accepted mediators of RANKL induction, did not inhibit *A. actinomycetemcomitans*-induced RANKL expression [18]. This demonstrates that the mechanism of RANKL induction by *A. actinomycetemcomitans* CDT is independent of classical inflammatory mediators. When purified *H. ducreyi* CDT was used as a model toxin to study RANKL induction in GF and PDL cells, it was alone able to induce RANKL, similarly to the wild-type *A. actinomycetemcomitans* strain. Alternatively, when it was added along with the CDT-mutant, it was able to rescue RANKL induction [45]. In another study employing T-cells, purified *H. ducreyi* CDT up-regulated RANKL gene expression and protein secretion, whereas OPG was not detected at all in this experimental system [46].

Collectively, CDT appears to induce RANKL, the key osteoclast-differentiating factor responsible for bone resorption, in several cell types of relevance to periodontal disease. Hence, this toxin of *A. actinomycetemcomitans* may be a crucial virulence factor for the pathological bone resorption occurring during the process of localized aggressive periodontitis.

**Table 1:** Summary of the literature: effects on cytolethal distending toxins (CDTs) on the production of host inflammatory mediators by various cell types.

*Species*	*Target cell*	*Effect*	*CDT-dependence*	*Reference*
*A. actinom.*	GF	IL-6 induction	Partly	[18]
	GF/PDL cells	RANKL induction	Yes	[45]
	PBMCs	IL-1β, IL-6, IL-8, IFN-γ induction	Yes	[11]
	Macrophages	NO, INF-γ inhibition	Yes	[17]
	MNL cells	NLRP3/NLRP6 regulation	No	[18]
*H. ducreyi*	GF/PDL cells	RANKL induction	Yes	[45]
	GF	IL-6 induction	Partly	[18]
	Various cell lines	IL-6, IL-8, IL-24 induction	Partly	[28]
	Dendritic cells	TNF-α, IL-6, IL-8, IL-12 inhibition	Partly	[29]
	Jurkat T-cells	RANKL induction	Yes	[46]
*C. jejuni*	Intestinal epithelium	IL-8 induction	Yes	[30]
	Colonic epithelium	IL-8 induction	Partly	[33]
*H. hepaticus*	Hepatic tissue	TNF-α, IFN-γ and IL-6	Yes	[40]

## 4. Conclusions

Several Gram-negative pathogenic species produce a CDT. Apart from the well accepted effects on inhibition of cell cycle and induction of apoptosis in host cells, this toxin can also affect the transcription or production of some inflammatory mediators in diverse cell types, and may involve the TLR/NFκ-B pathway. The CDT-producing species with documented such capacities are *A. actinomycetemcomitans*, *H. ducreyi*, *H. hepaticus* and *C. jejuni*, while the affected inflammatory mediators are primarily RANKL, IL-6 and IL-8. This could have implications on the pathogenesis of the associated infections. In terms of future research, novel transcriptomic or proteomic approaches may be able to shed further light onto the global effects of CDTs, with regards to inflammatory responses, and other aspects of CDT-related toxicity.

## References

[1] Guerra L., Cortes-Bratti X., Guidi R., Frisan T. (2011). The biology of the cytolethal distending toxins. Toxins (Basel).

[2] Heywood W., Henderson B., Nair S.P. (2005). Cytolethal distending toxin: Creating a gap in the cell cycle. J. Med. Microbiol..

[3] Guerra L., Guidi R., Frisan T. (2011). Do bacterial genotoxins contribute to chronic inflammation, genomic instability and tumor progression?. FEBS J..

[4] Smith J.L., Bayles D.O. (2006). The contribution of cytolethal distending toxin to bacterial pathogenesis. Crit. Rev. Microbiol..

[5] Kumar H., Kawai T., Akira S. (2011). Pathogen recognition by the innate immune system. Int. Rev. Immunol..

[6] Vladimer G.I., Marty-Roix R., Ghosh S., Weng D., Lien E. (2013). Inflammasomes and host defenses against bacterial infections. Curr. Opin. Microbiol..

[7] Johansson A. (2011). Aggregatibacter actinomycetemcomitans leukotoxin: A powerful tool with capacity to cause imbalance in the host inflammatory response. Toxins (Basel).

[8] Henderson B., Ward J.M., Ready D. (2010). Aggregatibacter (actinobacillus) actinomycetemcomitans: A triple a* periodontopathogen?. Periodontol 2000.

[9] Shenker B.J., McKay T., Datar S., Miller M., Chowhan R., Demuth D. (1999). Actinobacillus actinomycetemcomitans immunosuppressive protein is a member of the family of cytolethal distending toxins capable of causing a g2 arrest in human T cells. J. Immunol..

[10] Sato T., Koseki T., Yamato K., Saiki K., Konishi K., Yoshikawa M., Ishikawa I., Nishihara T. (2002). P53-independent expression of p21(cip1/waf1) in plasmacytic cells during g(2) cell cycle arrest induced by actinobacillus actinomycetemcomitans cytolethal distending toxin. Infect. Immun..

[11] Akifusa S., Poole S., Lewthwaite J., Henderson B., Nair S.P. (2001). Recombinant actinobacillus actinomycetemcomitans cytolethal distending toxin proteins are required to interact to inhibit human cell cycle progression and to stimulate human leukocyte cytokine synthesis. Infect. Immun..

[12] DiRienzo J.M., Song M., Wan L.S., Ellen R.P. (2002). Kinetics of kb and hep-2 cell responses to an invasive, cytolethal distending toxin-producing strain of actinobacillus actinomycetemcomitans. Oral Microbiol. Immunol..

[13] Belibasakis G., Johansson A., Wang Y., Claesson R., Chen C., Asikainen S., Kalfas S. (2002). Inhibited proliferation of human periodontal ligament cells and gingival fibroblasts by actinobacillus actinomycetemcomitans: Involvement of the cytolethal distending toxin. Eur. J. Oral Sci..

[14] Belibasakis G.N., Mattsson A., Wang Y., Chen C., Johansson A. (2004). Cell cycle arrest of human gingival fibroblasts and periodontal ligament cells by actinobacillus actinomycetemcomitans: Involvement of the cytolethal distending toxin. APMIS.

[15] Darveau R.P. (2010). Periodontitis: A polymicrobial disruption of host homeostasis. Nat. Rev. Microbiol..

[16] Lerner U.H. (2004). New molecules in the tumor necrosis factor ligand and receptor superfamilies with importance for physiological and pathological bone resorption. Crit. Rev. Oral Biol. Med..

[17] Fernandes K.P., Mayer M.P., Ando E.S., Ulbrich A.G., Amarente-Mendes J.G., Russo M. (2008). Inhibition of interferon-gamma-induced nitric oxide production in endotoxin-activated macrophages by cytolethal distending toxin. Oral Microbiol. Immunol..

[18] Belibasakis G.N., Johansson A., Wang Y., Chen C., Lagergard T., Kalfas S., Lerner U.H. (2005). Cytokine responses of human gingival fibroblasts to actinobacillus actinomycetemcomitans cytolethal distending toxin. Cytokine.

[19] Oscarsson J., Karched M., Thay B., Chen C., Asikainen S. (2008). Proinflammatory effect in whole blood by free soluble bacterial components released from planktonic and biofilm cells. BMC Microbiol..

[20] Belibasakis G.N., Johansson A. (2012). Aggregatibacter actinomycetemcomitans targets nlrp3 and nlrp6 inflammasome expression in human mononuclear leukocytes. Cytokine.

[21] Schroder K., Tschopp J. (2010). The inflammasomes. Cell.

[22] Mise K., Akifusa S., Watarai S., Ansai T., Nishihara T., Takehara T. (2005). Involvement of ganglioside gm3 in g(2)/m cell cycle arrest of human monocytic cells induced by actinobacillus actinomycetemcomitans cytolethal distending toxin. Infect. Immun..

[23] Akifusa S., Heywood W., Nair S.P., Stenbeck G., Henderson B. (2005). Mechanism of internalization of the cytolethal distending toxin of actinobacillus actinomycetemcomitans. Microbiology.

[24] Yamada T., Komoto J., Saiki K., Konishi K., Takusagawa F. (2006). Variation of loop sequence alters stability of cytolethal distending toxin (cdt): Crystal structure of cdt from actinobacillus actinomycetemcomitans. Protein Sci..

[25] Cao L., Bandelac G., Volgina A., Korostoff J., DiRienzo J.M. (2008). Role of aromatic amino acids in receptor binding activity and subunit assembly of the cytolethal distending toxin of aggregatibacter actinomycetemcomitans. Infect. Immun..

[26] Li L., Ding C., Duan J.L., Yang M.F., Sun Y., Wang X.Q., Xu Y. (2013). A new functional site w115 in cdta is critical for aggregatibacter actinomycetemcomitans cytolethal distending toxin. PLoS One.

[27] Hughes F.J., Turner W., Belibasakis G., Martuscelli G. (2006). Effects of growth factors and cytokines on osteoblast differentiation. Periodontol 2000.

[28] Blazkova H., Krejcikova K., Moudry P., Frisan T., Hodny Z., Bartek J. (2010). Bacterial intoxication evokes cellular senescence with persistent DNA damage and cytokine signalling. J. Cell. Mol. Med..

[29] Xu T., Lundqvist A., Ahmed H.J., Eriksson K., Yang Y., Lagergard T. (2004). Interactions of haemophilus ducreyi and purified cytolethal distending toxin with human monocyte-derived dendritic cells, macrophages and cd4+ T cells. Microbes Infect..

[30] Hickey T.E., McVeigh A.L., Scott D.A., Michielutti R.E., Bixby A., Carroll S.A., Bourgeois A.L., Guerry P. (2000). Campylobacter jejuni cytolethal distending toxin mediates release of interleukin-8 from intestinal epithelial cells. Infect. Immun..

[31] Li Y.P., Vegge C.S., Brondsted L., Madsen M., Ingmer H., Bang D.D. (2011). Campylobacter jejuni induces an anti-inflammatory response in human intestinal epithelial cells through activation of phosphatidylinositol 3-kinase/akt pathway. Vet. Microbiol..

[32] Elmi A., Watson E., Sandu P., Gundogdu O., Mills D.C., Inglis N.F., Manson E., Imrie L., Bajaj-Elliott M., Wren B.W. (2011). Campylobacter jejuni outer membrane vesicles play an important role in bacterial interactions with human intestinal epithelial cells. Infect. Immun..

[33] Zheng J., Meng J., Zhao S., Singh R., Song W. (2008). Campylobacter-induced interleukin-8 secretion in polarized human intestinal epithelial cells requires campylobacter-secreted cytolethal distending toxin- and toll-like receptor-mediated activation of nf-kappab. Infect. Immun..

[34] Gerondakis S., Fulford T.S., Messina N.L., Grumont R.J. (2014). Nf-kappab control of T cell development. Nat. Immunol..

[35] Jones M.A., Totemeyer S., Maskell D.J., Bryant C.E., Barrow P.A. (2003). Induction of proinflammatory responses in the human monocytic cell line thp-1 by campylobacter jejuni. Infect. Immun..

[36] Hickey T.E., Majam G., Guerry P. (2005). Intracellular survival of campylobacter jejuni in human monocytic cells and induction of apoptotic death by cytholethal distending toxin. Infect. Immun..

[37] Young V.B., Knox K.A., Pratt J.S., Cortez J.S., Mansfield L.S., Rogers A.B., Fox J.G., Schauer D.B. (2004). *In vitro* and *in vivo* characterization of helicobacter hepaticus cytolethal distending toxin mutants. Infect. Immun..

[38] Pratt J.S., Sachen K.L., Wood H.D., Eaton K.A., Young V.B. (2006). Modulation of host immune responses by the cytolethal distending toxin of helicobacter hepaticus. Infect. Immun..

[39] Ge Z., Feng Y., Whary M.T., Nambiar P.R., Xu S., Ng V., Taylor N.S., Fox J.G. (2005). Cytolethal distending toxin is essential for helicobacter hepaticus colonization in outbred swiss webster mice. Infect. Immun..

[40] Ge Z., Rogers A.B., Feng Y., Lee A., Xu S., Taylor N.S., Fox J.G. (2007). Bacterial cytolethal distending toxin promotes the development of dysplasia in a model of microbially induced hepatocarcinogenesis. Cell. Microbiol..

[41] Bostanci N., Ilgenli T., Emingil G., Afacan B., Han B., Toz H., Atilla G., Hughes F.J., Belibasakis G.N. (2007). Gingival crevicular fluid levels of rankl and opg in periodontal diseases: Implications of their relative ratio. J. Clin. Periodontol..

[42] Bostanci N., Ilgenli T., Emingil G., Afacan B., Han B., Toz H., Berdeli A., Atilla G., McKay I.J., Hughes F.J. (2007). Differential expression of receptor activator of nuclear factor-kappab ligand and osteoprotegerin mrna in periodontal diseases. J. Periodontal. Res..

[43] Bostanci N., Saygan B., Emingil G., Atilla G., Belibasakis G.N. (2011). Effect of periodontal treatment on receptor activator of nf-kappab ligand and osteoprotegerin levels and relative ratio in gingival crevicular fluid. J. Clin. Periodontol..

[44] Belibasakis G.N., Bostanci N. (2012). The rankl-opg system in clinical periodontology. J. Clin. Periodontol..

[45] Belibasakis G.N., Johansson A., Wang Y., Chen C., Kalfas S., Lerner U.H. (2005). The cytolethal distending toxin induces receptor activator of nf-kappab ligand expression in human gingival fibroblasts and periodontal ligament cells. Infect. Immun..

[46] Belibasakis G.N., Brage M., Lagergard T., Johansson A. (2008). Cytolethal distending toxin upregulates rankl expression in jurkat t-cells. APMIS.

